# Serum Level of miR-1 and miR-155 as Potential Biomarkers of Stress-Resilience of NET-KO and SWR/J Mice

**DOI:** 10.3390/cells9040917

**Published:** 2020-04-09

**Authors:** Joanna Solich, Maciej Kuśmider, Agata Faron-Górecka, Paulina Pabian, Magdalena Kolasa, Beata Zemła, Marta Dziedzicka-Wasylewska

**Affiliations:** Maj Institute of Pharmacology Polish Academy of Sciences, Smętna 12, 31-343 Kraków, Poland; kusmider@if-pan.krakow.pl (M.K.); gorecka@if-pan.krakow.pl (A.F.-G.); palach@if-pan.krakow.pl (P.P.); gaska@if-pan.krakow.pl (M.K.); zemla@if-pan.krakow.pl (B.Z.); marta.dziedzicka-wasylewska@uj.edu.pl (M.D.-W.)

**Keywords:** NET-KO mice, SWR/J mice, microRNA, stress-resilience, miR-1, miR-155, BDNF

## Abstract

In the present study, we used three strains of mice with various susceptibility to stress: mice with knock-out of the gene encoding norepinephrine transporter (NET-KO), which are well characterized as displaying a stress-resistant phenotype, as well as two strains of mice displaying two different stress-coping strategies, i.e., C57BL/6J (WT in the present study) and SWR/J. The procedure of restraint stress (RS, 4 h) was applied, and the following behavioral experiments (the forced swim test and sucrose preference test) indicated that NET-KO and SWR/J mice were less sensitive to RS than WT mice. Then, we aimed to find the miRNAs which changed in similar ways in the serum of NET-KO and SWR/J mice subjected to RS, being at the same time different from the miRNAs found in the serum of WT mice. Using Custom TaqMan Array MicroRNA Cards, with primers for majority of miRNAs expressed in the serum (based on a preliminary experiment using the TaqMan Array Rodent MicroRNA A + B Cards Set v3.0, Thermo Fisher Scientific, Waltham, MA, USA) allowed the identification of 21 such miRNAs. Our further analysis focused on miR-1 and miR-155 and their targets—these two miRNAs are involved in the regulation of BDNF expression and can be regarded as biomarkers of stress-resilience.

## 1. Introduction

Stress is defined as conditions that seriously perturb the psychological and physiological balance of an individual. However, the impact of stressful life events on physical and psychological well-being is highly variable, and does not affect everyone in the same manner; there are susceptible individuals who poorly adapt to stressors and express inappropriate responses that can become persistent states of stress, while resilient individuals can perceive adversity and develop adaptive psychological and physiological responses [1]. The underlying mechanisms of these responses are not fully understood as yet, although they are known to depend on a combination of genetic and non-genetic factors that interact in complex ways. Coping refers to alternative response patterns that occur in reaction to a challenging environment, and coping strategies are essential to minimize the impact of stress and determine the degree of resilience and susceptibility. However, as the biological basis of stress response is not clearly defined, the same concerns coping strategies [2].

The idea of microRNAs (miRNAs) as mediators of the brain genomic response to stress is quite new, although recently many studies have been devoted to this subject, and various miRNAs or miRNAs families have been shown to be regulated in response to different kinds of stress [3,4,5,6], and they are regarded as endogenous “hubs” (as Issler and Chen have put it) for the fine tuning of target gene expression or an “expression switch”, and can provide deeper insight into complex biological processes underlying stress response.

In the present study, we used three strains of mice differentially reacting to stress: mice with knock-out of the gene encoding norepinephrine transporter (NET-KO), which are well characterized as displaying a stress-resistant phenotype [7,8,9], as well as two other strains of mice displaying different stress-coping strategies, i.e., C57BL/6J (WT in the present study) and swiss SWR/J, described extensively by Szklarczyk and co-workers [10]. The different reaction to stress of these strains of mice has been confirmed by measuring corticosterone level. The NET-KO mice display a slower increase in corticosterone level after stress compared with WT animals, while corticosterone level was not changed at 2 h after restraint stress in the blood of SWR/J mice [8,10]. We applied the procedure of restraint stress (RS), which has been shown to induce an uncontrollable aversive situation that produces both physical and psychological consequences leading to neuronal and behavioral alterations [11]. The aim of following molecular studies was to find biomarkers for different behavioral responses to RS among miRNAs expressed in the serum of three genotypes under study. This goal was achieved, as we were able to identify a group of miRNAs differentiating the stress response of NET-KO and SWR/J mice from WT animals. The most interesting were the alterations in the miR-1 and miR-155 levels, which are involved in regulation of BDNF expression, which in turn affects important biochemical processes.

## 2. Materials and Methods

### 2.1. Animals

Heterozygous mice (C57BL/6J background), obtained from Dr M. Caron and generated by Xu and co-workers at Duke University Medical Center, Durham, NC, USA [12], were mated to each other. Homozygous WT and NET-KO (Slc6a2^tm1Mca^/IFPAS) males, ca. 4.5 month old, were used for the experiments. Genotypes were confirmed with a polymerase chain reaction (PCR) using the primers mNETEx2s (5′-GCT TTA TGG CAT GTA GTG TGC AC-3′), mNETEx2as (5′-GCT TTC TGC TTG AAC TTG AAG GC-3′), and EGFPas (5′-GCC GGA CAC GCT GAA CTT GTG-3′) to amplify 700 and 500 bp PCR products from WT and NET-KO mice, respectively.

The swiss (SWR/J) strain was obtained from the Jackson Laboratory (USA). Six week old males and females were imported to IFPAS and mated to each other. The 4.5 month old males were used for the experiments.

The mice were housed in groups. Animals had free access to food and water and were kept at a constant room temperature (24 °C) under a 12 h light/dark cycle. Animals were kept according to the decision of the Minister of Environment (no. 88/2014) and the Local Bioethic Commission (199/2017).

### 2.2. Behavioral Studies

#### 2.2.1. Restraint Stress (RS)

The mice were housed individually one week before the experiment. Ten male mice per group were used for behavioral tests. After the adaptation period, they were placed in well-ventilated polypropylene tubes (diameter 28 mm and length 110 mm), located in their home cage, for 4 h. During the immobilization phase, the mice did not have access to food and water, and after the experiment they were left undisturbed in their home cage.

#### 2.2.2. Forced Swim Test (FST)

The forced swim test was performed 6 days after the RS. Mice were individually placed in a transparent cylinder (20 cm in diameter) filled with water (23–25 °C) to a depth of 12 cm, to prevent them from using their tails to support themselves in the water. Immobility was measured during the last 4 min of a 6 min session. Immobility was defined as cessation of limb movements except for minor movements necessary to keep the mouse afloat [13].

#### 2.2.3. Sucrose Preference Test (SPT)

The sucrose preference test was performed 6 days after the RS. The experimental animals were acclimatized to two bottles of water one week before the RS; they were not deprived of food or water before the test. Then, they were given free choice between a 2% sucrose-solution and tap water for 24 h. The bottles with water and sucrose solution were replaced after 12 h of test time. The consumption of liquid was determined by weighting the bottles. The preference to sucrose was determined by comparison of sucrose-solution intake to the total amount of liquid drunk [14].

#### 2.2.4. Statistical Analysis of Behavioral Data

Data from behavioral experiments were analyzed with GraphPad Prism 7.04. Each genotype was investigated during separate experiments, therefore data were analyzed by the Student’s *t*-test to compare the stressed group with the control group of each genotype. Additionally, control groups of NET-KO and SWR/J were compared with the control group of WT mice. The Student’s *t*-test was also used for these comparisons. The *p* values less than 0.05 were considered significant.

### 2.3. Molecular Studies

#### 2.3.1. Blood Collection

The trunk blood was collected 10 min after the RS procedure from 7 male mice per group, as well as from mice not subjected to RS (control groups). Blood was left at room temperature for 20–30 min to clot. Then, it was centrifuged at 3000 rpm for 15 min at 4 °C. Serum was withdrawn and centrifuged again (3000 rpm for 5 min at 4 °C) to remove remaining cells. The hemolysis in serum was determined by the absorbance of hemoglobin at 414 nm using a NanoDrop ND-1000 (Thermo Fisher Scientific, Waltham, MA, USA). Serum samples with absorbance ≤ 0.3 were considered as unhemolyzed [15], and stored at −80 °C until further purification.

#### 2.3.2. Isolation of miRNAs from Serum

Total RNAs, including small RNAs, were isolated from 80 µL of the serum using miRNeasy Serum/Plasma Kit (Qiagen, Germantown, MD, USA) according to the manufacturer’s instructions. We used 1 µg MS2 RNA as a carrier to increase recovery. The synthetic spiked-in ath-miR-159a was added to each sample at the amount of 3 × 10^9^ copies to monitor RNA extraction [16]. The 400 µL of QIAzol lysis reagent and 80 µL of chloroform were added to each sample. The recovered amount of aqueous phase was always 240 µL. The 360 µL of 100% ethanol was added per sample. The whole sample was transferred to the column, which was washed according to the manual’s instructions. The 14 µL RNase-free water was used to elute the RNA from the column. The quality and quantity of the isolated total RNA were evaluated by a NanoDrop ND-1000 (Thermo Fisher Scientific) and Experion microcapillary electrophoresis system (Bio-Rad). Samples that passed the quality threshold (RIN > 8.0) were used for further experiments.

#### 2.3.3. miRNA RT-qPCR Array

A preliminary experiment using the TaqMan Array Rodent MicroRNA A + B Cards Set v3.0 (Thermo Fisher Scientific was performed to determine serum expressed miRNAs. The set enables accurate quantitation of 641 unique microRNAs. The primers for 191 miRNAs expressed in the serum of all experimental groups of mice as well as the primers for ath-miR-159a (used later for normalization) were placed on the Custom TaqMan Array MicroRNA Cards used for the main experiment. One Custom TaqMan Array MicroRNA Card was used per sample. The samples were ran in duplicates. The cDNA was synthetized by a TaqMan MicroRNA Reverse Transcription Kit (Thermo Fisher Scientific) with a Megaplex RT Primers Rodent Pools Set v3.0—preliminary experiment—or with a TaqMan Custom RT Pool for further experiments, according to the manufacturer’s instructions. The obtained cDNA was preamplified to increase the quantity of desired cDNA using a TaqMan PreAmp Master Mix (Thermo Fisher Scientific) with Megaplex PreAmp Primers—preliminary experiment—or TaqMan Custom PreAmp Primers for further studies. The preamplified product was diluted and RT-qPCR reactions were performed with the TaqMan Universal PCR Master Mix, No AmpErase UNG (Thermo Fisher Scientific). The qPCRs were ran on a QuantStudio 12K Flex System (Applied Biosystems, Waltham, MA, USA).

Data were further analyzed with the QuantStudio 12K Flex Software (Applied Biosystems). A Ct value above 30 was considered as undetectable miRNAs due to the preamplification. The same threshold equal to 0.2 was set for all samples for comparison. Then, the data were analyzed with qBasePLUS 3.1 software (Biogazelle, Zwijnaarde, Belgium) which enables whole genome miRNA profiling with global mean normalization. The miRNAs like mmu-miR-301a-3p and mmu-miR-7a-1-3p—suitable for normalization—were generated by the geNorm algorithm [17,18]. Afterwards, statistical analysis was carried out with GraphPad Prism 7.04 by two-way ANOVA (Tukey’s post-test for multiple comparisons between groups) to compare each miRNA expression changes between genotypes under control conditions and following RS. A value of *p* ≤ 0.05 was considered to be significant.

#### 2.3.4. Identification of miRNA Targets In Silico

The miRNAs with expression level significantly differentiating the experimental groups of animals were subjected to further bioinformatics analyses. The DIANA-miRPath v.3.0, an online software, was used for the assessment of miRNA regulatory roles and the identification of controlled pathways [19]. Following pathway enrichment analysis using Fisher’s Exact Test (hypergeometric distribution), the miRNAs targets were selected from TarBase v7.0. A value of *p* ≤ 0.05 was considered to be significant and FDR correction was selected.

Additionally, the miRNet v1.0—the integrated platform linking miRNAs, targets and functions—was used to find a connection network of miRNAs and target genes regulated by them [20]. Only genes regulated by two or more miRNAs were indicated, therefore degree cutoff equal to 1 was selected from the degree filter. Then, the results of the computational approaches were validated experimentally on the mRNA level.

#### 2.3.5. Isolation of mRNAs from the Liver

The livers of four mice of each genotype under the control and stress conditions were separated 10 min after RS, frozen and stored at −80 °C until further use. The TRI Reagent (Sigma-Aldrich, New York, NY, USA) was used for RNA purification, according to manufacturer’s instructions. The liver was homogenized with 1 mL of reagent per sample, in the homogenizer stirrer (Glas-Col, Terre Haute, IN, USA; 099C, K4424). The amount of aqueous phase was always 400 µL. The 30 µL of RNase-free water was used for dissolved the RNA pellet. The quality and quantity of the isolated total RNA were evaluated by a NanoDrop ND-1000 (Thermo Fisher Scientific) and Experion microcapillary electrophoresis system (Bio-Rad, Hercules, CA, USA). Samples that passed the quality threshold (RIN > 8.0) were used for further experiments.

#### 2.3.6. Quantitative RT-qPCR Analyses of Individual mRNAs

The RNA was reverse transcribed to cDNA transcripts according to the manufacturer’s protocol, using a High Capacity cDNA Reverse Transcription Kit (Thermo Fisher Scientific). For RT-qPCR reactions, the TaqMan Universal Master Mix II (no UNG) and TaqMan Gene Expression Assay (Thermo Fisher Scientific) were added to the 20 ng of cDNA template. The list of TaqMan Gene Expression Assays was included in the Supplementary Materials (Table S1). The real-time PCR reactions were carried out in duplicates using a CFX96 Touch Real-Time PCR Detection System (Bio-Rad) with the following cycles: enzyme activation at 95 °C for 10 min, 40 cycles of denaturation at 95 °C for 15 s, and subsequent annealing/elongation at 60 °C for 60 s. The results were normalized to β-actin (Actb) and glyceraldehyde-3-phosphate dehydrogenase (Gapdh). Then, the results were analyzed with CFX Manager Software (Version 3.1; Bio-Rad) using the ∆∆Cq method to obtain the relative normalized expression values. GraphPad Prism (Version 7.04) was used for statistical analysis. Two-way ANOVA was used to determine the statistical significance of the differences. The mRNA expression levels were compared by genotype and stress response. These analyses were followed by Tukey’s post-test for multiple comparisons between groups. The *p*-values ≤ 0.05 were considered statistically significant.

#### 2.3.7. Glucose Measurement

The trunk blood glucose level was measured in 8–9 male mice per group using a Diagnostic Gold glucometer (Diagnosis SA, Białystok, Poland), 10 min after RS. The statistical analysis was performed by one-way ANOVA with GraphPad Prism (Version 7.04) to determine the differences between groups. The *p*-values ≤ 0.05 were considered statistically significant.

## 3. Results

Six groups of animals were studied: three different genotypes (WT, NET-KO, SWR/J) were compared under control conditions and following restraint stress (RS). The forced swim test (FST) and sucrose preference test (SPT) were conducted in different cohorts of animals.

### 3.1. Behavioral Studies

Behavioral studies were performed in order to confirm the differences in vulnerability to stress of the three studied genotypes. Animals (control and subjected to RS) were exposed to the forced swim test (FST) and sucrose preference test (SPT), and the obtained results indicated that the response to stress varied between genotypes, and even a delayed effect of single RS was observed.

**Figure 1:**
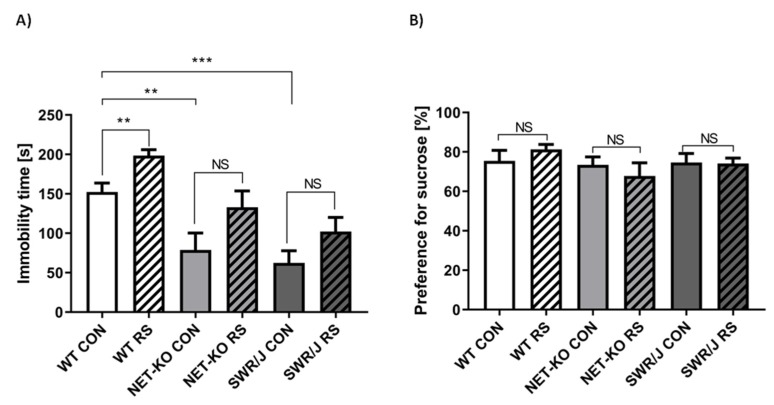
Results of the behavioral tests: the forced swim test (FST; (**A**)), the sucrose preference test (SPT; (**B**)). The immobility time or preference for sucrose of wild-type (WT), norepinephrine transporter knock-out (NET-KO) and swiss (SWR/J) mice during FST or SPT was measured under the control conditions (CON) and 6 days after restraint stress (RS). Each genotype was investigated during separate experiments. ** *p* ≤ 0.01; *** *p* ≤ 0.001; NS—not significant; *n* = 10.

As indicated by the FST, NET-KO and SWR/J mice displayed shorter immobility time than WT mice, which is usually interpreted as depression-resistant behavior, since a similar reaction is observed upon antidepressant drugs administration to WT animals [7]. Single RS induced even longer immobility time in WT animals, while this effect was not significant in NET-KO as well as SWR/J mice (Figure 1A), indicating that these two genotypes were somehow resilient to RS, as far as their reaction in the FST was concerned.

The preference for sucrose in all studied genotypes was not different either under the control conditions or 6 days after single RS (Figure 1B).

### 3.2. Molecular Studies

The aim of our molecular studies was to find biomarkers for different behavioral responses to RS among miRNAs expressed in the serum of the three genotypes under study. For this reason, we first determined the miRNAs that are expressed in the serum of all three genotypes under the control and stress conditions. Out of 768 miRNAs present on both cards of the TaqMan Array Rodent MicroRNA, there were 146 miRNAs expressed on the A Card, while 56 miRNAs were expressed on the B Card. The list of miRNAs has been included in the Supplementary Materials (Table S2). Then, the Custom TaqMan Array MicroRNA Cards were used, with primers for majority of miRNAs expressed in the serum.

#### 3.2.1. Control Condition

##### NET-KO Mice vs. WT Mice

(1) KEGG Pathway Analysis

**Figure 2:**
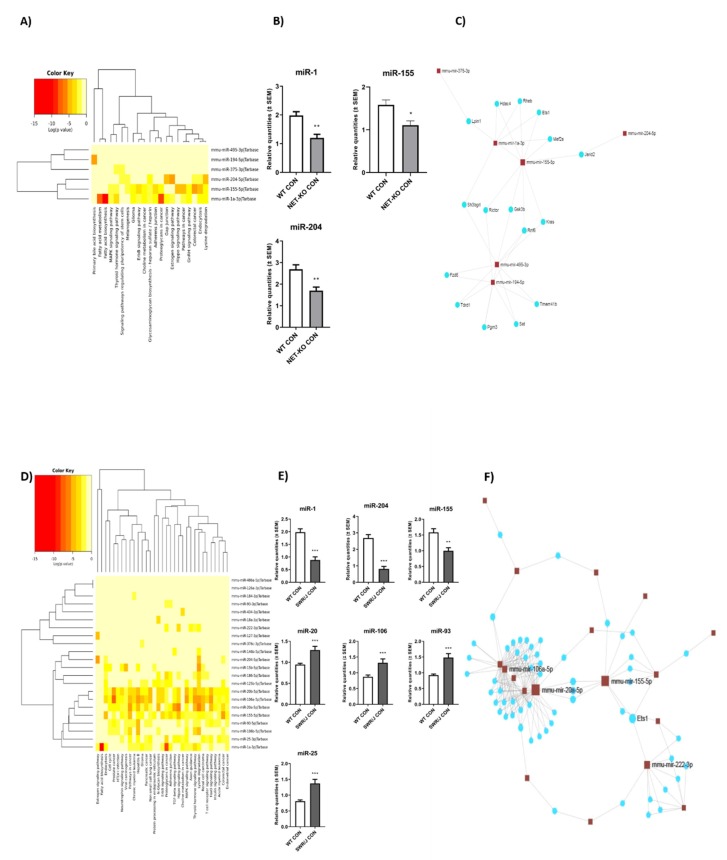
The expression of miRNAs in the serum of three genotypes of mice under the control condition. Panels (**A**–**C**) show the results of the comparison of norepinephrine transporter knock-out (NET-KO) with WT mice. Panels (**D**–**F**) show the results of the comparison of SWR/J with WT mice. The heatmaps show the miRNAs and KEGG pathways relationship (**A**,**D**). The miRNAs are placed on the right side, while the KEGG pathways are placed at the bottom of the map. The significance of linking miRNA to KEGG category is color-coded (the scale in the upper, left corner). Panels (**B**,**E**) represent the expression alterations of miRNAs displaying the strongest connection to KEGG pathways. Panels (**C**,**F**): the connection networks of miRNAs and target genes (details in Table S3). ** *p* ≤ 0.01; *** *p* ≤ 0.001; *n* = 7.

The eight miRNAs differentiated NET-KO from WT mice. Six of them were present in the TarBase v7.0 using KEGG pathway analysis with DIANA-miRPath v.3.0. These miRNAs were found to regulate genes associated with 21 pathways (Table S4). The most interesting of these pathways were the following: the thyroid hormone signaling pathway, fatty acid metabolism and biosynthesis as well as Gap junction, endocytosis or lysine degradation (Figure 2A).

(2) Analysis of Clustered miRNAs Expression

The miR-204, miR-155 and miR-1, which were clustered together, are mainly involved in these pathways’ regulation, and their expression was lower in the serum of NET-KO in comparison to WT mice (Figure 2B). The effect of genotype on the changes of miRNAs expression was significant (miR-1 (F (2,32) = 15.68; *p* < 0.0001); miR-155 (F(2,35) = 8.47; *p* = 0.001); miR-204 (F (2,36) = 34.20; *p* < 0.0001).

(3) Linking miRNAs to Target Genes—miRNet Network

The miRNAs differentiating NET-KO from WT mice were then linked to target genes (in such a way that they indicated genes regulated by at least two miRNAs), which resulted in the network linking all six miRNAs with the relevant genes. Among them were: Hdac4, Rheb, Ets1, Mef2a regulated by miR-1 and miR-155, as well as Gsk3b regulated by miR-155 and miR-195 (Figure 2C).

##### SWR/J Mice vs. WT Mice

(1) KEGG Pathway Analysis

On the other hand, there were 25 miRNAs which differentiated SWR/J from WT mice. One miRNA was not found in the TarBase v7.0. The remaining miRNAs were clustered into three groups. One of them showed a more significant relationship to the KEGG pathways. This group included miR-1, miR-20, miR-106, miR-155, miR-93 and miR-25. The miR-204 also differentiated SWR/J from WT mice, but it was positioned in another cluster. The following signaling pathways are regulated by these miRNAs: fatty acid biosynthesis, neurotrophin, TGF-beta, thyroid hormone, and insulin as well as lysine degradation, endocytosis and MAPK signaling pathway (Figure 2D; Table S4).

(2) Analysis of Clustered miRNAs Expression

The expression of miR-1, miR-204 and miR-155 was decreased in the serum of SWR/J mice as compared to the control group of WT animals. However, the expression of the remaining miRNAs was higher when SWR/J mice were compared to WT control group (Figure 2E). The effect of genotype on the changes of miRNAs expression was significant (miR-1 (F (2,32) = 15.68; *p* < 0.0001); miR-204 (F (2,36) = 34.20; *p* < 0.0001); miR-155 (F(2,35) = 8.47; *p* = 0.001); miR-20 (F(2,36) = 27.57; *p* < 0.0001); miR-106 (F(2,35) = 17.52; *p* < 0.0001); miR-93 (F(2,36) = 40.68; *p* < 0.0001); miR-25 (F(2,36) = 48.42; *p* < 0.0001).

(3) Linking miRNAs to Target Genes—miRNet Network

The linking miRNAs which differentiated SWR/J from WT mice with the target genes (regulated by at least two of them) resulted in the network (Figure 2F), including 18 miRNAs and 58 genes. Among them were miR-1, miR-20, miR-106, miR-155, miR-222, miR-204 as well as some interesting genes, like Il10rb, Timp2, Stat3, Hdac4, Mapk14 and Kcnk6. The list of miRNAs and their target genes is included in the Supplementary Materials (Table S3).

#### 3.2.2. Stress Condition (Restraint Stress, RS)

The behavioral experiments described above indicated that NET-KO and SWR/J mice were less sensitive to RS than WT mice. Therefore, this part of the study aimed to find the miRNAs which changed in similar ways in the serum of NET-KO and SWR/J mice subjected to RS, being at the same time different from the miRNAs found in the serum of WT mice.

##### KEGG Pathway Analysis

**Figure 3:**
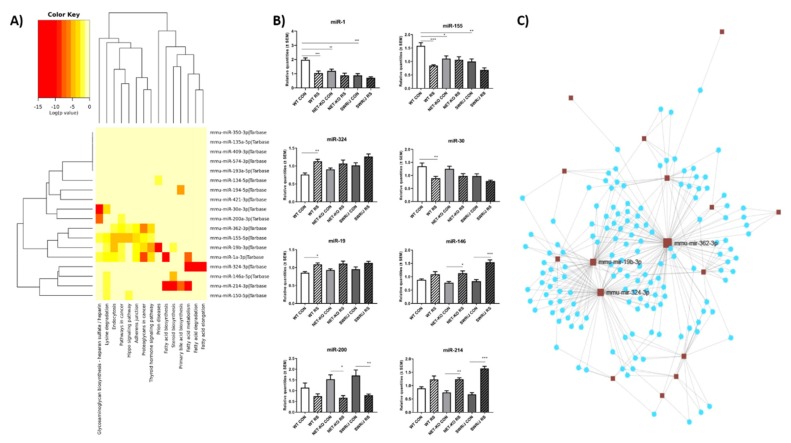
The expression of miRNAs in the serum of three genotypes of mice following restraint stress (RS). The heatmap shows miRNAs differentiating norepinephrine transporter knock-out (NET-KO) and SWR/J mice from WT animals following RS, in relation to KEGG pathways (**A**). The miRNAs are placed on the right side, while the KEGG pathways are placed at the bottom of the map. The significance of linking miRNA to KEGG category is color-coded (the scale in the upper, left corner). Panel (**B**) shows expression alterations of miRNA displaying the strongest connection to KEGG pathways. Panel (**C**)—the connection networks of miRNAs and target genes (details in Table S3). * *p* ≤ 0.05, ** *p* ≤ 0.01; *** *p* ≤ 0.001; *n* = 7. Statistical differences not marked on the graphs on Panel (**B**): miR-1 (WT CON vs. NET-KO RS *p* ≤ 0.001; WT CON vs. SWR/J RS *p* ≤ 0.001); miR-155 (WT CON vs. NET-KO RS *p* ≤ 0.01; WT CON vs. SWR/J RS *p* ≤ 0.001; NET-KO CON vs. SWR/J RS *p* ≤ 0.05); miR-324 (WT CON vs. NET-KO RS *p* ≤ 0.05; WT CON vs. SWR/J RS *p* ≤ 0.001; NET-KO CON vs. SWR/J RS *p* ≤ 0.01); miR-30 (WT CON vs. SWR/J RS *p* ≤ 0.001; NET-KO CON vs. SWR/J RS *p* ≤ 0.01); miR-19 (WT CON vs. NET-KO RS *p* ≤ 0.01; WT CON vs. SWR/J RS *p* ≤ 0.01); miR-146 (WT CON vs. SWR/J RS *p* ≤ 0.001; NET-KO CON vs. WT RS *p* ≤ 0.05; NET-KO CON vs. SWR/J RS *p* ≤ 0.001; WT RS vs. SWR/J RS *p* ≤ 0.01; NET-KO RS vs. SWR/J RS *p* ≤ 0.01); miR-200 (NET-KO CON vs. WT RS *p* ≤ 0.05; NET-KO CON vs. SWR/J RS *p* ≤ 0.05; SWR/J CON vs. WT RS *p* ≤ 0.01; SWR/J CON vs. NET-KO RS *p* ≤ 0.01); miR-214 (WT CON vs. NET-KO RS *p* ≤ 0.05; WT CON vs. SWR/J RS *p* ≤ 0.001; NET-KO CON vs. WT RS *p* ≤ 0.01; NET-KO CON vs. SWR/J RS *p* ≤ 0.001; SWR/J CON vs. WT RS *p* ≤ 0.001; SWR/J CON vs. NET-KO RS *p* ≤ 0.001; WT RS vs. SWR/J RS *p* ≤ 0.01; NET-KO RS vs. SWR/J RS *p* ≤ 0.05).

We were able to identify 21 miRNAs that changed in similar ways in the serum of NET-KO and SWR/J mice subjected to RS. Three of them were not found in TarBase v7.0. The remaining ones are described as affecting the regulation of pathways related to biosynthesis, metabolism, elongation and degradation of fatty acids; steroid biosynthesis; endocytosis and lysine degradation, as well as the thyroid hormone signaling pathway (Figure 3A; Table S4). The miR-1, miR-324, miR-214, miR-19, miR-30, miR-200, miR-155 and miR-146 were the most related to these pathways. They were combined in three clusters: one cluster included miR-1, miR-19 and miR-155, second one—miR-324, miR-146 and miR-214, and the third one included miR-30 and miR-200.

##### Analysis of Clustered miRNAs Expression

The alterations in expression of miRNAs mentioned above under RS conditions are shown in Figure 3B. Some miRNAs (miR-1, miR-155, miR-324, miR-30 and miR-19) were differently expressed only in the serum of WT mice following RS conditions, but they were stable in the serum of NET-KO and SWR/J mice after stress. On the other hand, the expression of miR-146, miR-200 and miR-214 was significantly changed in the serum of NET-KO and SWR/J after RS, but it was not altered in the WT animals. The effect of RS on the changes of miRNAs expression was significant (miR-1 (F(1,32) = 20.09; *p* < 0.0001); miR-155 (F(1,35) = 22.88; *p* < 0.0001); miR-324 (F (1,36) = 22.63; *p* < 0.0001); miR-30 (F(1,36) = 17.93; *p* = 0.0002); miR-19 (F(1,36) = 23.89; *p* < 0.0001); miR-146 (F(1,36) = 50.44; *p* < 0.0001); miR-200 (F(1,33) = 27.95; *p* < 0.0001); miR-214 (F(1,36) = 85.43; *p* < 0.0001).

##### Linking miRNAs to Target Genes—miRNet Network

The miRNAs differentiating the stress response of NET-KO and SWR/J mice from WT animals were connected in the network if mRNA was regulated by more than one miRNA. This network included 16 miRNAs and 148 mRNAs (Figure 3C). The list of miRNAs and their target genes has been included in the Supplementary Materials (Table S3). The mRNA targets for this group of miRNAs were connected with the maintenance of homeostasis and response to stress, ion binding, biosynthetic processes as well as chromosome organization. Some of them were nucleic acid binding transcription factors.

#### 3.2.3. Verification of miR-1 Related Gene Expression

**Figure 4:**
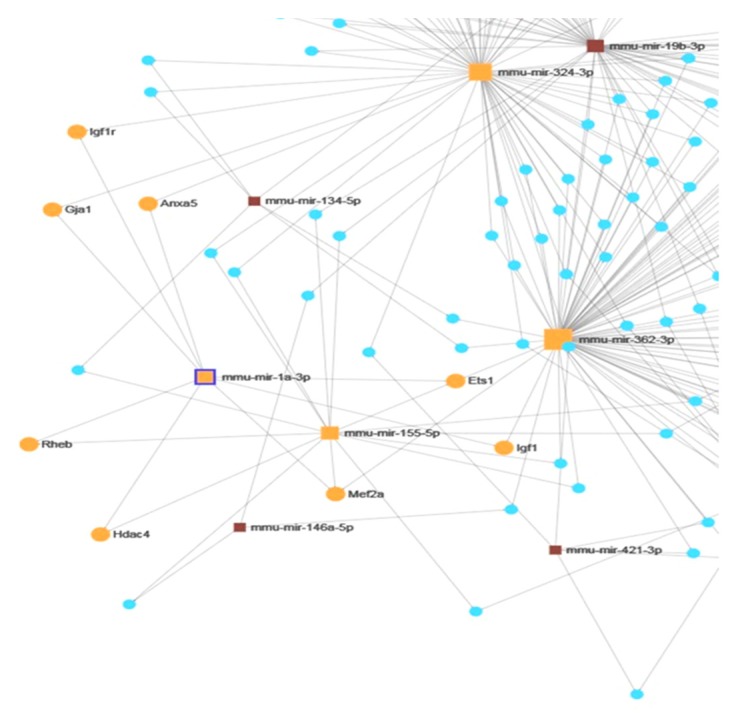
Connection network of miRNAs and their target genes differentiating the stress reactions of norepinephrine transporter knock-out (NET-KO) and SWR/J mice from WT animals, focused on miR-1-regulated genes (orange). The squares represent miRNAs while the circles represent the mRNAs.

Our further analysis focused on miR-1, because it differentiated control groups of NET-KO as well as SWR/J from WT mice, and also it differentiated the stress response of NET-KO and SWR/J mice from WT animals. Analyses described above pointed to the following mRNAs regulated by miR-1: Igf1, Igf1r, Hdac4, Mef2a, Anxa5, Rheb, Ets1, and Gja1. The miR-1 connected network is shown in Figure 4.

The expression of some genes, indicated by miRNet analysis as regulated by miR-1, was checked in the liver. Additionally, BDNF and Ntrk2 were also selected, based on the available literature.

**Figure 5:**
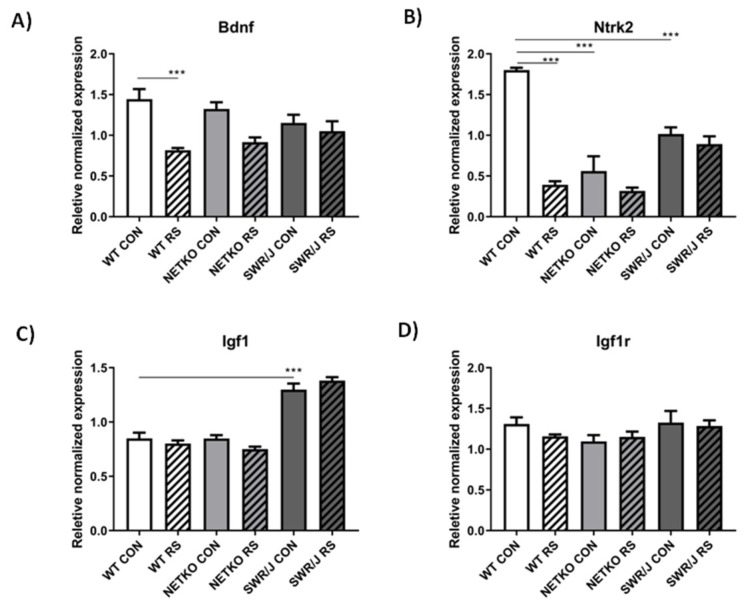
The expression of mRNA encoding the genes regulated by miR-1 in the liver of wild-type (WT), norepinephrine transporter knock-out (NET-KO) and swiss (SWR/J) mice under the control condition (CON) and after stress (RS). Brain derived neurotrophic factor (BDNF; (**A**)); neurotrophic tyrosine kinase, receptor, type 2 (Ntrk2; (**B**)); insulin-like growth factor 1 (Igf1; (**C**)); insulin-like growth factor 1 receptor (Igf1r; (**D**)). *** *p* ≤ 0.001; *n* = 4. Statistical differences not marked on the graphs: A) WT CON vs. NET-KO RS *p* ≤ 0.01; WT CON vs. SWR/J RS *p* ≤ 0.05; NET-KO CON vs. WT RS *p* ≤ 0.01; B) WT CON vs. NET-KO RS *p* ≤ 0.001; WT CON vs. SWR/J RS *p* ≤ 0.001; NET-KO CON vs. SWR/J CON *p* ≤ 0.05; SWR/J CON vs. WT RS *p* ≤ 0.001; SWR/J CON vs. NET-KO RS *p* ≤ 0.001; WT RS vs. SWR/J RS *p* ≤ 0.01; NET-KO RS vs. SWR/J RS *p* ≤ 0.001; C) WT CON vs. SWR/J RS *p* ≤ 0.001; NET-KO CON vs. SWR/J CON *p* ≤ 0.001; NET-KO CON vs. SWR/J RS *p* ≤ 0.001; SWR/J CON vs. WT RS *p* ≤ 0.001; SWR/J CON vs. NET-KO RS *p* ≤ 0.001; WT RS vs. SWR/J RS *p* ≤ 0.001; NET-KO RS vs. SWR/J RS *p* ≤ 0.001.

The expression of mRNA encoding BDNF in all three genotypes did not differ under control conditions (Figure 5A), however it was reduced after RS in the liver of WT mice, while it did not change in other genotypes. The effect of stress on mRNA expression was significant (F(1,40) = 24.20; *p* < 0.0001), while the effect of genotype was insignificant (F(2,40) = 0.045; *p* = 0.95). In turn, expression of mRNA encoding Ntrk2 was significantly higher in the WT mice as compared to the control group of NET-KO and SWR/J mice, and it was reduced following RS in the WT mice, while it did not change in the liver of NET-KO and SWR/J mice after stress (Figure 5B). The effect of stress on mRNA expression was significant (F(1,32) = 64.65; *p* < 0.0001), and the effect of genotype was also significant (F(2,32) = 30.64; *p* < 0.0001). In the SWR/J mice, a significantly higher level of Igf1 mRNA was observed in comparison with both WT and NET-KO mice, and no differences in this mRNA level were observed following RS in all genotypes (Figure 5C). The effect of genotype on the changes of mRNA expression was significant (F(2,42) = 121.3; *p* < 0.0001), while the effect of stress was insignificant (F(1,42) = 0.39; *p* = 0.53). As far as the expression of mRNA encoding for Igf1 receptor (Igf1r)—no differences were observed between the studied genotypes both under the control and stress conditions (Figure 5D).

#### 3.2.4. Glucose Measurement

**Figure 6:**
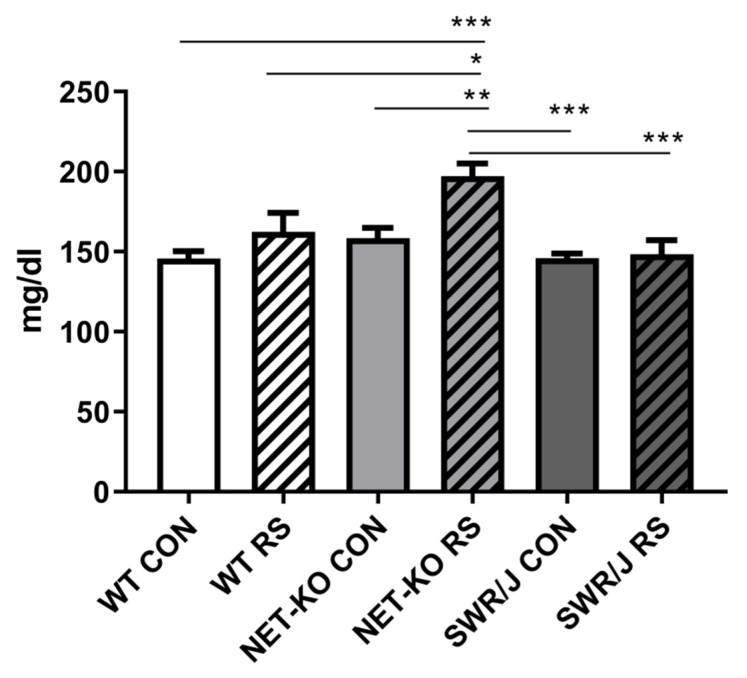
The level of glucose measured in the blood of wild-type (WT), norepinephrine transporter knock-out (NET-KO) and swiss (SWR/J) mice under the control condition (CON) and after stress (RS). * *p* ≤ 0.05, ** *p* ≤ 0.01; *** *p* ≤ 0.001; *n* = 8–9.

Glucose level was not different in the blood of WT, NET-KO and SWR/J mice under the control condition. Following RS, it was increased, but only in the blood of NET-KO mice (Figure 6).

## 4. Discussion

The results obtained in the present study indicate that varied behavioral responses to the stress of mice depended on the genotype and was accompanied by changes in the expression of some miRNAs in the serum. Immobility time during the forced swim test (FST) of the control NET-KO mice was significantly reduced compared with WT control animals. This result correlated with previously published data [7,8,12,21]. In turn, the immobility time of WT mice increased after RS but did not change significantly when NET-KO mice subjected to RS were compared with the NET-KO control group. The same effect has been shown by Haenisch and colleagues following 21 days of RS [9]. On the other hand, there was no change in the sucrose preference test (SPT) at 6 days after RS. In the previous study, it has been shown that NET-KO mice displayed greater preference for the sucrose solution, which did not change after stress in contrast to WT mice [9]. Perhaps the difference in sugar content in the solution consumed by the mice affected this effect. The NET-KO mice are known to display a stress-resilient phenotype, and this effect has been linked to an elevated level of norepinephrine, as shown by Isingrini and co-workers [22]. Likewise, SWR/J mice exhibited a stress-resilient phenotype as demonstrated in the present study (reduced immobility time of SWR/J compared to WT under the control condition and no changes after RS). The results are in agreement with the data published previously [10]. In turn, the lack of any effect in the SPT might result from a different flavor preference of SWR/J mice [23].

Since it has been postulated that the stress response may be associated with miRNAs present in the serum, we decided to look for markers of stress-resilience in three genotypes of mice responding differently to RS. Therefore, our preliminary molecular studies identified miRNAs, which were expressed in the serum of all three groups of mice. Most of the miRNAs detected in the present study have been also identified by Mi and co-workers [24]. Further studies of miRNAs expression under control conditions allowed for determination of the basal level of various miRNAs. During this part of the study, we also identified those miRNAs that differentiated both the NET-KO and SWR/J control mice from WT animals. Three miRNAs are interesting—miR-1, miR-155 and miR-204. These miRNAs are connected with proliferation, apoptosis, and cardiac injury [25] as well as transforming growth factor-β1 (TGF-β1) and glial cell line-derived neurotrophic factor (GDNF) regulation during glaucoma, asthma and obesity [26,27,28,29]. However, what is even more interesting is that it has been also shown that these miRNAs regulate brain-derived neurotrophic factor (BDNF) [30,31,32]. Other miRNAs differentiating the SWR/J control from the WT control group of mice, such as miR-20 and miR-106, are associated with the regulation of glucose metabolism [33], whilst miR-93 and miR-106 might affect liver diseases [34]. Additionally, miR-106 has been linked with regulation of the MAPK signaling pathway during oxidative stress [35]. The differences in expression of these miRNAs in the serum of NET-KO, SWR/J and WT mice under control conditions may affect the subsequent genotype dependent stress response.

Among miRNAs that differentiated the response to stress of NET-KO and SWR/J mice from WT animals were miR-1 and miR-155, mentioned earlier, but also miR-324, miR-30, miR-19, miR-146, miR-200 and miR-214. The changes in miR-324 and miR-19 expression occurred only in the serum of WT mice under stress conditions, while in other genotypes they did not differ. This effect is particularly interesting, since these miRNAs have been shown as associated with post-traumatic stress disorder (PTSD): the studies by Balakathiresan and colleagues on the animal model of PTSD identified miR-324 and miR-19 as differentially expressed when compared with the serum of control animals, which correlated with the expression of these miRNAs in the amygdala [36]. Another interesting miRNA identified in the present study is miR-30, which was downregulated following RS in the serum of WT mice but not in the NET-KO and SWR/J animals. The alterations in miR-30 level were also described after social defeat stress in the dentate gyrus of the C57BL/6 mice [37]. These results suggest that the abovementioned changes may be associated with the stress-susceptible phenotype of WT mice, while no change was found in the stress-resilient phenotypes, i.e., NET-KO and SWR/J mice. Next, interesting miRNA found in the present study is miR-146, alterations of which are associated with immune system response or with prolonged exercise [31,38], but there have been no reports so far about association of this miRNA with stress. In turn, miR-200 was sensitive to oxidative stress and modulated endothelial inflammation [39], but also regulated glucose levels in diabetes [40]. It could be suggested that expression alteration of miR-200 in the serum of NET-KO and SWR/J mice under stress conditions could be linked with alterations in the blood level of glucose. Our studies also showed changes in miR-214 expression under stress conditions, but only in NET-KO and SWR/J mice. This miRNA has been associated with regulation of diabetes [41], but other studies have shown its role in the regulation of stress (chronic social defeat stress), mediated by β-catenin [42].

All the miRNAs mentioned above are interesting in the context of resilience to RS, however, the most interesting results obtained in the present study concern miR-1. The level of this miRNA was lower in the serum of control groups of NET-KO and SWR/J, and it decreased in WT mice upon RS, while it did not change in other two genotypes. The miR-1 has been associated with cardiac dysfunction, immunology (allergy), skeletal muscle changes (amyotrophic lateral sclerosis), but also as a regulator of glucose metabolism in the liver [25,43,44,45]. It was also detected during stress studies, such as temperature changes, exercise (longer running), and stress connected with elevated platforms [38,46,47,48]. Only longer exercises increased miR-1 expression, while long-term temperature stress and single stress in the elevated platform apparatus caused a decrease in expression of this miRNA. Eivani and co-workers have shown a decrease of miR-1 expression in the rat brain, which was associated with decreased BDNF level [48]. It demonstrates that the decrease in mRNA expression is not always accompanied by an increase in miRNA expression. This is probably due to the influence of other factors. In turn, Sun and colleagues demonstrated changes in the liver and linked them with glucose metabolism [45]. Studies on cerebral palsy also proved miR-1 regulation of mRNA encoding BDNF [49], and it has been also shown that miR-1 prevented apoptosis via Igf1 regulation [50]. In the present study, we were able to identify certain genes regulated by miR-1, as shown in the network connections (Figure 4), and—following the data provided by papers cited above—we additionally focused on expressions of BDNF and its receptor (Ntrk2). We chose the mouse liver because it was evidenced that mRNAs encoding both Igf1 and BDNF were found in this organ [51,52]. Although the expression of most mRNAs was not changed, we have demonstrated alterations in expressions of BDNF and Ntrk2 in response to RS in WT mice. The result is interesting in the context of the data provided by Hsu and co-workers [53], who have shown that bipolar disorder patients develop liver disease more often, while Yang and colleagues found a negative correlation between mature BDNF in the brain and liver of depressed patients [54]. Additionally, these results are even more interesting if one takes into account that miR-1 and miR-155, described above as differentiating the response to stress of NET-KO and SWR/J mice from WT animals, regulate the expression of the gene encoding BDNF [30]. It correlates with data provided by Camer and co-workers, who have shown that the reduction of BDNF-TrkB signaling could result from a decrease of the BDNF level in the liver [55]. In turn, Tonra and colleagues presented evidence that BDNF improved blood glucose control [56]. In the present study, we show that glucose levels increased significantly in NET-KO mice subjected to RS, and—besides indicating again that the stress response varied between WT and NET-KO mice—such effect might result from the alterations in miRNAs involved in glucose regulation (miR-1, miR-155 and miR-200), shown as important markers of stress-resilience in the present study. This effect was not observed in the SWR/J mice, which may be associated with higher Igf1 mRNA expression in the liver of these mice compared with WT and NET-KO mice.

In conclusion, our research indicates that the varied response to stress depended on the genotype, and the serum level of miR-1 and miR-155, which are involved in regulation of BDNF expression, can be regarded as biomarkers of stress-resilience.

## Supplementary Materials

The following are available online at https://www.mdpi.com/2073-4409/9/4/917/s1, Table S1: List of TaqMan Gene Expression Assays added to RT-qPCR reactions, Table S2: The list of miRNA expressed on the TaqMan Array Rodent MicroRNA A+B Cards Set v3.0 during preliminary experiments, Table S3: The list of miRNAs differentiating studied groups of mice and their target genes. Wild-type (WT), norepinephrine transporter knock-out mice (NET-KO), swiss mice (SWR/J), control condition (CON), stress condition (RS), Table S4: The list of KEGG Pathways regulated by miRNAs differentiating studied groups of mice. Wild-type (WT), norepinephrine transporter knock-out mice (NET-KO), swiss mice (SWR/J), control condition (CON), stress condition (RS).
