# Supplementary material for: Serum Level of miR-1 and miR-155 as Potential Biomarkers of Stress-Resilience of NET-KO and SWR/J Mice
Source: Cells. 2020 Apr 9;9(4):917. doi: 10.3390/cells9040917 (PMC7226811; doi:10.3390/cells9040917)
Supplement: Supplementary file 1 [file cells-09-00917-s001.zip › Solich et al supplementary materials/Table S1.docx]

Table S1

| **Target name** | **Gene** | **Assay ID** |
| --- | --- | --- |
| insulin-like growth factor 1 | Igf1 | Mm00439560_m1 |
| insulin-like growth factor I receptor | Igf1r | Mm00802831_m1 |
| brain derived neurotrophic factor | Bdnf | Mm01334042_m1 |
| neurotrophic tyrosine kinase, receptor, type 2 | Ntrk2 | Mm00435422_m1 |
| glyceraldehyde-3-phosphate dehydrogenase | Gapdh | Mm99999915_g1 |
| actin, beta | Actb | Mm00607939_s1 |
|  |  |  |
|  |  |  |
